# GLIS1-3: Links to Primary Cilium, Reprogramming, Stem Cell Renewal, and Disease

**DOI:** 10.3390/cells11111833

**Published:** 2022-06-03

**Authors:** Anton M. Jetten, David W. Scoville, Hong Soon Kang

**Affiliations:** Cell Biology Section, Immunity, Inflammation and Disease Laboratory, National Institute of Environmental Health Sciences, National Institutes of Health, Research Triangle Park, Durham, NC 27709, USA; david.scoville@nih.gov (D.W.S.); kang1@niehs.nih.gov (H.S.K.)

**Keywords:** GLIS Krüppel-like zinc finger protein, transcription, stem cells, primary cilium, self-renewal, breast cancer, leukemia, liver fibrosis, circGLIS, reprogramming

## Abstract

The GLI-Similar 1-3 (*GLIS1-3*) genes, in addition to encoding GLIS1-3 Krüppel-like zinc finger transcription factors, also generate circular GLIS (circGLIS) RNAs. GLIS1-3 regulate gene transcription by binding to GLIS binding sites in target genes, whereas circGLIS RNAs largely act as miRNA sponges. GLIS1-3 play a critical role in the regulation of many biological processes and have been implicated in various pathologies. GLIS protein activities appear to be regulated by primary cilium-dependent and -independent signaling pathways that via post-translational modifications may cause changes in the subcellular localization, proteolytic processing, and protein interactions. These modifications can affect the transcriptional activity of GLIS proteins and, consequently, the biological functions they regulate as well as their roles in disease. Recent studies have implicated GLIS1-3 proteins and circGLIS RNAs in the regulation of stemness, self-renewal, epithelial-mesenchymal transition (EMT), cell reprogramming, lineage determination, and differentiation. These biological processes are interconnected and play a critical role in embryonic development, tissue homeostasis, and cell plasticity. Dysregulation of these processes are part of many pathologies. This review provides an update on our current knowledge of the roles GLIS proteins and circGLIS RNAs in the control of these biological processes in relation to their regulation of normal physiological functions and disease.

## 1. Introduction

**Figure 1:**
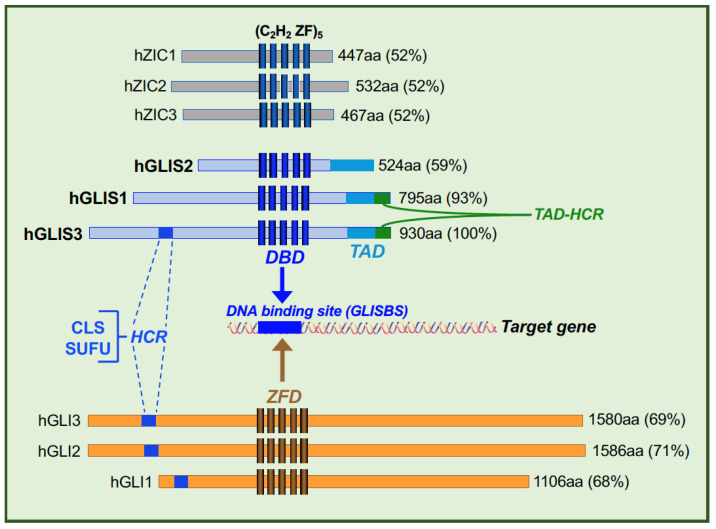
GLIS1-3 proteins are most closely related to members of the GLI subfamily Krüppel-like zinc finger transcription factors. The DNA binding domain (DBD) consists of five C_2_H_2_ zinc fingers that exhibit high similarity with those of GLI1-3 and several ZIC family members. The DBD recognizes a GC-rich GLIS binding site (GLISBS) in regulatory region of target genes. Shown in parenthesis is the percent homology of each DBD relative to GLIS3-DBD. The transactivation domain (TAD) at the C-terminus of GLIS1 and GLIS3 contains a 32 amino acids sequence that is highly conserved (TAD-CR). The N-terminus of GLIS3 and GLI1-3 share a highly conserved region (HCR) of about 60 amino acids that contains a SUFU binding site and a ciliary localization signal (CLS) [6,15].

GLI-Similar 1-3 (GLIS1-3) constitute a distinct subgroup of Krüppel-like zinc finger proteins that function as activators and repressors of gene transcription [1,2,3,4,5,6,7]. GLIS proteins contain a highly conserved DNA binding domain (DBD) consisting of five Cys_2_His_2_ zinc finger motifs that bind to GLIS binding sites, referred to as GLISBS, consisting of a G-rich consensus sequence in the regulatory regions of target genes (Figure 1). The DBD of GLIS3 exhibits a 93% and 54% homology with that of GLIS1 and GLIS2, respectively, and a 68–71% similarity with those of GLI1-3, Krüppel-like zinc finger proteins involved in hedgehog pathway-dependent transcriptional regulation. The GLIS3-DBD exhibits a 52% homology with that of members of the ZIC family of Krüppel-like zinc finger proteins. Because of the high homology in their DBDs, members of the GLI and ZIC subfamilies recognize DNA-binding sequences that are very similar to those of GLISBS. It is, therefore, likely that members of these families compete for the same G-rich binding site and thereby interfere with each other’s regulation of gene transcription and function. This is supported by data showing that GLIS2 inhibits GLISBS-dependent transcriptional activation by GLI1 [8] and the GLI1-induced transcriptional activation of *Snai1* and Wingless 4 (*Wnt4*) by competing for the same binding site [9]. The transcriptional activation by GLIS1-3 is mediated by the recruitment of co-activators, such as EP300/p300, to the transactivation domain (TAD) at their carboxy-terminus (Figure 1). Although GLIS2 can activate gene transcription, in several cell types it appears to function largely as a repressor [3,5,8,10,11,12,13]. GLIS2 repressor function is, in part, mediated through its recruitment of the co-repressor, C-terminal binding protein 1 (CTBP1) [5,14]. Outside their DBDs, GLIS proteins exhibit little sequence homology with each other or with GLI and ZIC proteins, with two exceptions. GLIS3 and GLIS1 share a highly conserved 32 amino acid sequence (60% homology) within their TAD (referred to as TAD-HCR) that may be important for the interaction with common co-activators (Figure 1). The N-terminus of GLIS3 further contains a region of about 60 amino acids, referred to as highly conserved region (HCR), that exhibits high homology with a region present in the N-terminus of all three GLI proteins (Figure 1).

Study of loss-of-GLIS-function mutations in humans and *Glis* knockout mice showed that impairments in GLIS protein function are causally linked to the development of many pathologies [6,7,16]. Lack of GLIS3 is implicated in several diseases, including neonatal diabetes, congenital hypothyroidism, male infertility, polycystic kidney disease, and glaucoma [17,18,19,20,21,22,23,24,25]. Deficiency in GLIS2 causes nephronophthisis, a cystic renal disease that is the most common cause of end-stage renal disease in young adults [8,12,26,27], while GLIS1 was found to play a critical role in the regulation of intraocular pressure by maintaining normal trabecular meshwork functions [28]. GLIS1-3 have also been implicated in several malignancies, including breast and colorectal cancer, and leukemia [11,16,29,30,31,32,33,34]. A translocation involving CBFA2/RUNX1 Partner Transcriptional Co-Repressor 3 (*CBFA2T3*, also referred to as *ETO2*) and *GLIS2* has been implicated in acute mega-karyoblastic leukemia, while *GLIS1-Paired* Box 8 *(PAX8)* and *GLIS3-PAX8* translocations are associated with hyalinizing trabecular tumors, a rare thyroid neoplasm stemming from thyroid follicular cells [13,16,33,35,36,37,38].

Stem cell renewal, EMT, mesenchymal epithelial transition (MET), cell reprogramming, lineage determination, and differentiation are interrelated biological processes that play a key role in embryonic development, tissue homeostasis, and cell plasticity [39]. Dysregulation of these processes are causally involved in many diseases. Recent studies have demonstrated that GLIS proteins can regulate stem cell renewal, reprogramming, EMT, and cell lineage determination and that in certain cases this involves the primary cilium [11,13,40,41,42,43,44]. In this review, we focus particularly on several recent studies that identified roles for GLIS1-3 and circGLIS RNAs in the regulation of stem cell renewal, EMT, differentiation, and reprogramming, and their relevance to several diseases, including cancer. In addition, we discuss established links between these interrelated biological processes, primary cilium-associated pathways, and the regulation of GLIS transcriptional activity and function. Greater insights into the mechanisms by which GLIS1-3 regulate gene transcription and physiological functions will provide a better understanding of their roles in disease and might lead to the discovery of new therapeutic strategies in the management of these diseases.

## 2. GLIS Proteins and Primary Cilium

Immunostaining of various tissues and cultured cells have localized GLIS3 largely to the nucleus; however, GLIS3 has also been detected in the tip of primary cilia [18,45]. GLIS3 localization to the primary cilium is supported by mass spectrometric analysis of primary cilia-associated proteins [46]. Whether there is any connection between GLIS1 and the primary cilium, has yet to be established. Phylogenetic analyses of *GLIS1*/3 genes have provided certain clues for potential roles of both GLIS1 and GLIS3 in primary cilium-dependent functions [47]. The primary cilium is a microtubule-based, non-motile sensory organelle protruding from the plasma membrane of most cell types [48,49,50,51]. Primary cilia play a critical role in mediating the activation of several signaling pathways by a wide range of external signals, such as Wingless (WNT) proteins, insulin growth factor 1 (IGF1), platelet-derived growth factor α (PDGFα), hedgehogs, and mechanical stress, and therefore in the regulation of many cellular functions and biological processes, including cell fate specification, proliferation, and stemness [51,52,53]. Defects in primary cilium assembly or primary cilium-associated signaling pathways are causally linked to several pathologies, collectively referred to as ciliopathies that include polydactyly, nephronophthisis, and polycystic kidney disease [49,51,54,55,56].

The interaction of external signals with their respective receptors (e.g., G protein-coupled receptors, GPCRs) in the ciliary membrane result in changes in the activity specific ciliary proteins, including several protein kinases and transcription factors [49,50]. This might involve changes in ciliary cAMP and Ca^2+^ levels, post-translational modifications, proteolytic processing, and protein–protein interactions as demonstrated for GLI proteins. The formation of the primary cilium is cell cycle-dependent; the primary cilium is formed in G0/G1 interphase of the cell cycle and disassembled before cell division. Transport of proteins in and out the primary cilium is mediated by the intraflagellar transport system (IFT) [50]. The transition zone (TZ) at the base of the primary cilium forms a barrier between the cytoplasm and primary cilium and controls the entry and exit of proteins, including that of GLI proteins [57,58,59]. The N-terminus of GLI1-3 contains a ciliary localization signal (CLS) with the consensus sequence SSXR-X6-R/KKR-X5-PY/L containing an Arg/Lys-rich nuclear localization-like signal [50,58,60,61]. Transportin (TNPO1, also referred to as karyopherin β2 or importin 2), interacts with the CLS and mediates the entry of GLI1-3 into the primary cilium. A consensus CLS is also present within the 60 amino acid HCR at the N-terminus of GLIS3 [6,7,62] (Figure 1). We hypothesize that TNPO1 can also mediate the entry of GLIS3 into the primary cilium and that GLIS3-mediated transcriptional regulation is mediated by a primary cilium-dependent (canonical) pathway via a still to be identified primary cilium-associated GPCR. However, GLIS3 activity might also be controlled by a primary cilium-independent (noncanonical) mechanism. In addition to the CLS, the HCR contains a consensus binding site (ΦYGHΦ) for suppressor of fused (SUFU), a protein with multiple roles in hedgehog signaling that has also been localized to the primary cilium [63] (Figure 1). SUFU has been reported to interact with GLIS3 and to protect it from proteosomal degradation [62].

GLIS2 also has been localized to primary cilia and shown to interact with SUFU [9,12]. However, GLIS2 does not contain a consensus CLS sequence suggesting that its ciliary localization might be mediated by a different mechanism. Regulation of GLIS2 transcriptional activity by primary cilium-associated mechanisms is discussed below in more detail [11,44].

GLIS proteins are post-translationally modified by phosphorylation, sumoylation, ubiquitination, and acetylation, and they can undergo proteolytic cleavage [9,11,62,64,65,66]. Although the roles of many of these post-translational modifications are still largely unknown, they can alter the subcellular localization, protein stability, proteolytic processing, and transcriptional activity of GLIS proteins and, consequently, affect the regulation of their biological functions, as shown for GLIS2 and GLIS3 [11,44,62,66].

## 3. GLIS1-3: Regulation of Self-Renewal and EMT in Relation to Tumorigenesis

*GLIS1-*3 have been implicated in various malignancies, including leukemia, thyroid cancer, and breast cancer (reviewed in [16]). Dysregulation of EMT and stem cell renewal play critical roles in promoting cancer and tumor progression by inhibiting cell differentiation and increasing cell proliferation, migration, and metastasis. In several GLIS-associated malignancies, GLIS proteins have been reported to regulate stem cell renewal, EMT, and/or reprogramming in several tumor cell types [11,13,33,35,67,68].

**Figure 2:**
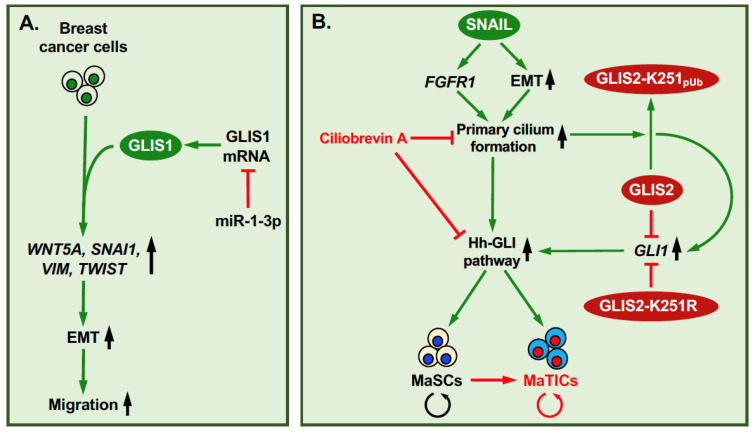
Roles of GLIS1 and GLIS2 in breast cancer. (**A**) Expression of GLIS1 in breast cancer cells increases the expression of EMT promoting genes, such as *SNAI1*, *VIM*, *WNT5A*, and *TWIST*, thereby promoting cell migration. MiR-1-3p inhibits the expression of GLIS1 and EMT. (**B**) Schematic of the interplay between SNAIL, primary cilium, regulation of GLIS2 repressor activity, and stem cell renewal. SNAIL induces GLIS2 polyubiquitination at K251 through a primary cilium-dependent mechanism that involves stimulation of EMT and FGFR1 expression. This polyubiquitination causes loss of GLIS2 repressor function, and subsequent activation of *GLI1* and *WNT* genes and increased stem cell renewal [11]. These actions are inhibited by ciliobrevin A, a ciliogenesis and hedgehog pathway inhibitor. GLIS2-K251R functions as a constitutive repressor and inhibitor of stem cell renewal.

Breast cancer is heterogeneous disease comprised of multiple tumor subtypes, including triple-negative (estrogen receptor-, progesterone receptor-, and epidermal growth factor receptor 2-negative; ER^−^PR^−^HER2^−^) breast tumors. All three *GLIS* genes have been linked to breast cancer. GLIS3 is expressed at significantly higher levels in breast cancer samples than in normal tissue and is particularly elevated in triple-negative breast tumors [30]. In MMTV-Cut like homeobox 1 (CUX1) transgenic mice, a mouse model used to study breast cancer development, *GLIS1* was found to be highly expressed in a subset of breast tumors with elevated *WNT* expression [69]. This correlated with increased β-catenin transcriptional activity and expression of epithelial–mesenchymal transition (EMT)-promoting genes, such as snail family transcriptional repressor 1 (*SNAI1*), vimentin (*VIM*), and twist family BHLH transcription factor 1 (*TWIST*)*,* and enhanced cell migration and invasion (Figure 2A). Similarly, hypoxia-induced GLIS1 expression in MDA-MB-231 breast carcinoma cells caused an increase in WNT5A expression and cell migration [31]. A separate study reported that expression of microRNA miR-1-3p suppressed EMT and cell migration in MDA-MB-231 cells by binding to the 3′-UTR of *GLIS1* mRNA resulting in reduced GLIS1 expression [70]. These studies suggest that GLIS1 plays a critical role in promoting EMT reprogramming in breast cancer cells.

A recent study identified an important role for GLIS2 in the regulation of stemness in multipotent mammary stem cells (MaSCs) and mammary tumor-initiating cells (MaTICs) [11]. MaTICs and cells from claudin-low breast tumors, which represent a subtype of triple-negative tumors thought to stem from MaTICs, exhibit many of the stem cell properties of MaSCs (Figure 2B). In addition to their self-renewal ability, MaSCs can differentiate into luminal and basal progenitors that, subsequently, give rise to ductal luminal and alveolar cells, and myoepithelial cells, respectively. As such, MaSCs play a critical role in coordinating mammary gland homeostasis, morphogenesis, and alveologenesis during embryonic development as well as postnatally [71]. Activation of the EMT program promotes and maintains the self-renewal capacity of MaSCs by inducing the formation of primary cilia that is mediated in part via increased expression of FGFR1 by EMT transcription factors, such as SNAIL2 (also known as SLUG) (Figure 2B) [52,72]. This, subsequently, leads to the primary cilium-dependent activation of the hedgehog-GLI1 signaling pathway and increased self-renewal [50,52,73]. Loss of primary cilia represses hedgehog signaling and, subsequently, reduces self-renewal in MaSCs and MaTICs, thereby reducing the tumor-producing potential of MaTICs.

The transcription factor SNAIL2 plays a critical role in the activation of EMT in both MaSCs and MaTICs [11,74]. This is accompanied by increased expression of EMT marker genes, such as N-cadherin (*CDH2*) and *VIM*, and the transcription factors, Zinc finger E-box binding homeobox 1 (*ZEB1*) and *TWIST*, and reduced expression of several epithelial markers, including E-cadherin (*CDH1*). The study by Wilson et al. [11] further demonstrated that GLIS2 represses MaSC self-renewal, whereas inactivation or loss of GLIS2 function promotes MaSC self-renewal indicating that GLIS2 functions as a repressor of MaSC stemness by directly suppressing the transcription of *GLI1* and *WNT* genes (Figure 2B). Based on these findings one might predict that GLIS2 deficiency would promote expansion of MaSCs at the cost of their differentiation into different mammary gland lineages and result in defective mammary gland development. This hypothesis was supported by observations showing that Glis2-deficient mice develop only small mammary rudiments [11]. A SNAIL-dependent post-translational modification of GLIS2 was found to play a crucial role in the regulation of self-renewal of MaSCs by GLIS2. SNAIL stimulates GLIS2 polyubiquitination at K251 leading to loss of GLIS2 repressor function (Figure 2B). The latter was not due to increased proteolytic degradation or reduced *GLIS2* mRNA expression, but to the loss of GLIS2 repressor activity [11]. Ciliobrevin A, an inhibitor of ciliogenesis, inhibited GLIS2 polyubiquitination and the activation of the hedgehog signaling pathway. Although the precise molecular mechanism has yet to be elucidated, these data indicate that the regulation of GLIS2 polyubiquitination by SNAIL is primary cilium-dependent (Figure 2B). The importance of GLIS2 polyubiquitination is supported by data showing that the K251R mutation converts GLIS2 into a constitutive repressor and consequently abrogates the SNAIL-induced expression of GLI1 and CDH11 in mammary cells [11].

*GLIS2* has also been implicated in the regulation of self-renewal in leukemic cells [16,32,33,34,68,75,76,77,78]. Several studies demonstrated that a cryptic inversion of chromosome 16 that fuses *CBFA2T3* to *GLIS2*, is frequently (25–30%) associated with a pediatric non-Down’s syndrome (non-DS) acute megakaryoblastic leukemia (AMKL) with poor prognosis [32,34,75,76,77,78,79,80]. Transcriptomic analysis indicated that CBFA2T3-GLIS2 regulates gene transcription partly through a CBFA2T3- or GLIS2-mediated mechanisms, but that a subset of genes are regulated via a CBFA2T3-GLIS2-specific mechanism. Among the genes regulated by CBFA2T3-GLIS2 were those with established roles in leukemia and hematopoietic stem cell self-renewal and the control of EMT and cell migration, including genes linked to the NOTCH, sonic hedgehog (SHH), WNT, hippo, and TGFβ/BMP signaling pathways [13,33,35,80,81]. Both the CBFA2T3 and GLIS2 domains are required to optimally stimulate hematopoietic stem cell self-renewal. CBFA2T3-GLIS2 was found to induce activation of super enhancers that regulate genes critical in leukemia [81]. It was further shown that CBFA2T3-GLIS2 greatly increased the expression of the ETS transcription factor ERG, a strong inducer of hematopoietic stem cell renewal, in a GLIS2-dependent manner and down-regulated the expression of GATA binding protein 1 (GATA-1), a promoter of megakaryocytic differentiation [13]. The role of CBFA2T3-GLIS2 in promoting self-renewal was supported by a recent study showing increased self-renewal by CBFA2T3-GLIS2 in a human induced pluripotent stem cells-derived model [67]. A study of murine MOZ-TIF2 acute myelogenous leukemia (AML) cells revealed that GLIS2 expression suppressed leukemic stem cell self-renewal and promoted differentiation [68].

## 4. GLIS2, Primary Cilium, PROM1 and Cell Renewal

**Figure 3:**
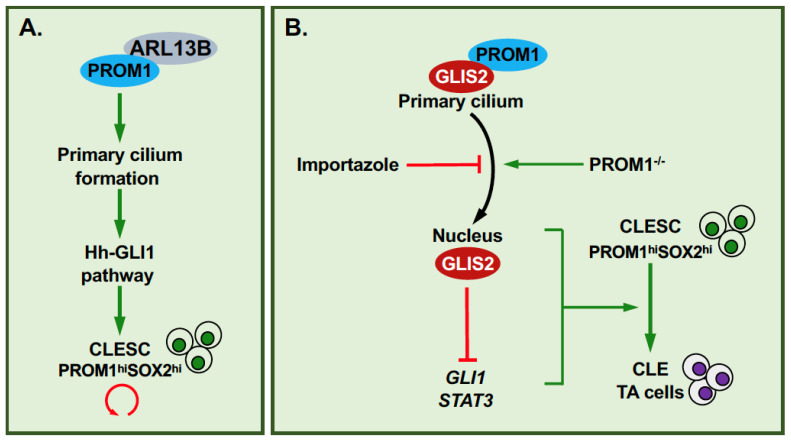
Link between PROM1, GLIS2, and stem cell renewal. (**A**) The interaction of PROM1 with the ciliary protein ARL13B promotes primary cilium formation and elongation leading to increased activation of the hedgehog (Hh)-GLI signaling pathway and stem cell renewal [84]. (**B**) GLIS2 interacts with PROM1 and localizes to the primary cilium. Loss of PROM1 promotes nuclear localization of GLIS2 where it represses the transcription of target genes, including *GLI1* and *STAT3,* thereby inhibiting CLESC renewal and promoting their differentiation into CLE transient amplifying cells (TA) [44].

A recent study identified a link between GLIS2, the primary cilium, prominin-1, and cell renewal [44]. Prominin-1 (PROM1/CD133) is a cholesterol-binding, pentaspan membrane glycoprotein, associated with several plasma membrane protrusions, including primary cilia [44,82]. PROM1 has been implicated in the regulation of stem cell maintenance, differentiation, and cancer [44,82,83]. In addition, PROM1 plays a role in the regulation of ciliary dynamics and the length and function of primary cilia through its interaction with the ciliary protein, ADP-ribosylation factor-like protein 13B (Arl13b) (Figure 3A) [44,84]. Study of the incisor cervical loop epithelium (CLE) showed that PROM1^hi^SOX2^hi^ CLE stem cells (CLESCs) have longer cilia than PROM1^lo^Ki67^hi^ CLE transit amplifying cells and that loss of PROM1 function inhibits primary cilium elongation, disrupts primary cilium-associated signaling pathways, including sonic hippo (SHH) pathway activation, and significantly reduces stem cell renewal (clonogenic capacity)(Figure 3B) [44]. These observations indicated a link between PROM1, primary cilium dynamics, and the regulation of stem cell self-renewal by SHH. This study further showed that the transition of CLESCs into CLE transit amplifying cells is accompanied by a decrease in Gli1-3 mRNA expression and an increase in Glis2 mRNA expression, and a translocation of GLIS2 and PROM1 proteins from the primary cilium to the nucleus. This translocation was inhibited by importazole, an inhibitor of importin β1-mediated nuclear import. In *Prom1*-KO mice, GLIS2 was largely localized to the nucleus suggesting that PROM1 is required for the localization of GLIS2 to the primary cilium, while in *Glis2*-KO mice PROM1 expression and the number of SOX2^+^ stem cells were shown to be significantly reduced, indicating an interrelationship between GLIS2 and PROM1 in regulating stemness. Together, these observations led to the hypothesis that translocation of GLIS2 to the nucleus during the transition of stem cell to transit amplifying cells allows it to function as a transcription repressor, thereby reducing the expression of genes (e.g., *Gli1*, *Stat3*) that are critical for stem cell maintenance and renewal (Figure 3B) [44]. The inhibition of stem cell renewal by GLIS2 involves repression of the transcription activation of *Stat3*, a GLIS2 target gene, and inhibition of GLI1-mediated transcriptional activation due to competition between GLIS2 and GLI1 for binding to the same enhancer in target genes. The inhibition of CLESC renewal is consistent with the function of GLIS2 in stem cell renewal reported for MaSCs [11]. Interestingly, PROM1 has been reported to be also expressed in MaSCs and MaTICs [85] suggesting that it might play a similar role in these cells as in CLESCs.

## 5. Effects of GLIS1-3 on iPSC Reprogramming and Differentiation

Several studies have demonstrated that GLIS1-3 can modulate reprogramming of somatic cells into induced pluripotent stem cells (iPSCs) [43,86,87,88,89,90,91,92,93]. Over-expression of the transcription factors, octamer-binding protein 4 (OCT4 or POU5F1), SRY-box transcription factor 2 (SOX2), Krüppel-like zinc finger protein 4 (KLF4), and c-MYC (OSKM) induces reprogramming of somatic cells into iPSCs [94]. Reprogramming of fibroblasts into iPSCs requires mesenchymal–epithelial transition (MET) that is mediated in part through the repression the EMT-inducing genes *SNAIL* by SOX2 and OCT4, and *TGFB1* and *TGFBR2* by c-MYC, and induction of MET-associated genes, such as E- cadherin (*CDH1*) by KLF4 [95]. Over-expression of GLIS1 in human and mouse fibroblasts markedly enhanced reprogramming efficiency by either OSK or OSKM, whereas down-regulation of endogenous GLIS1 expression reduced reprogramming efficiency by OSK [43,88,96]. However, in human adipose-derived stromal cells the GLIS1-mediated increase in reprogramming was dependent on co-expression with c-MYC [87]. A recent study showed that over-expression of GLIS1 synergized with Nanog homeobox (NANOG) in improving reprogramming efficiency, but not with LIN28, and that efficiency was reduced when GLIS1 was combined with NANOG and LIN28 [86]. In human fibroblasts, GLIS1 enhanced the expression of several reprogramming-promoting genes, including estrogen receptor-related receptor B (*ESRRB*), *LIN28A*, *NANOG*, *FOXA2*, *MYCL1*, *MYCN*, and several *WNT* genes. The induction of *ESRRB*, *LIN28A*, *NANOG*, and *FOXA2* was mediated through an indirect mechanism, whereas *MYCL1* and *MYCN* were directly regulated by GLIS1 [42]. GLIS1 regulates gene transcription in coordination with other transcription factors, including OCT4, SOX2, and KLF4. GLIS1 was found to interact with KLF4 and both its ZFD and N-terminus were required for this interaction. Together these studies indicate that GLIS1 enhances reprogramming efficiency by stimulating MET and activating the canonical WNT signaling pathway. A different study showed that expression of GLIS1, Spalt like transcription factor 4 (SALL4), liver receptor homolog 1 (LRH1) in combination with Jun dimerization protein 2 (JDP2), lysine demethylase 2B (KDM2B or JHDM1B), and ID1 induce reprogramming of fibroblasts into iPSCs, but that GLIS1 was not required [43,97]. GLIS1 was reported to be highly expressed in unfertilized eggs and one-cell embryos, but at very low levels in blastocysts and pluripotent stem cells (PSCs) [42] suggesting that it does not play a significant role in maintaining pluripotency in PSCs in vivo.

In addition to GLIS1, GLIS3 has also been shown to enhance reprogramming efficiency. Co-expression of GLIS3 was shown to promote reprogramming of human adipose-derived stromal cells by OSKM as efficiently as GLIS1 [87]. In contrast to GLIS1 and GLIS3, co-expression of GLIS2 inhibited reprogramming [87]. However, a different study reported that GLIS2 knockdown in hPSCs repressed the expression of pluripotency genes, such as *OCT4* and *SOX2*, and promoted their differentiation into endodermal and trophoblast lineages suggesting that GLIS2 expression maintains stemness of hPSCs [98]. Future studies are needed to explain these apparent contrasting effects of GLIS2 on reprogramming and hESC stemness.

In the effects of GLIS proteins on reprogramming, one needs to consider that KLF4 and GLIS proteins bind similar G-rich binding sequences suggesting that there might be some overlap between target genes they regulate. This is supported by a study showing that GLIS1 can substitute for KLF4 in reprogramming [88]. This appears to be supported by a recent report indicating that GLIS1 and KLF4 share some common functions and/or mechanisms in reprogramming [91]. In the case of GLIS2, by functioning as a repressor GLIS2 might also compete with KLF4 for binding and as a result suppress the activation of certain KLF4 target genes and reduce reprogramming efficiency.

## 6. GLIS2 and Reprogramming in Hepatic Fibrosis

**Figure 4:**
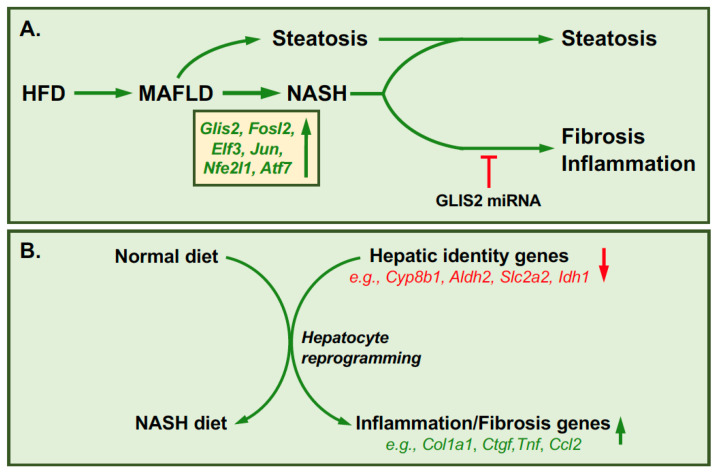
Role of GLIS2 in hepatocyte reprogramming during non-alcoholic steatohepatitis (NASH). (**A**) Diet-induced MAFLD can progress to the development of NASH. This is accompanied by increased expression of several transcription factors, including GLIS2, FOSL2, JUN, ATF7, NFE2L1, and ELF3, that together regulate the induction of inflammatory and fibrotic genes [41]. Down-regulation of GLIS2 by Glis2-miRNA inhibits inflammation and fibrosis but has no effect on steatosis (lipid accumulation). (**B**) Hepatocyte reprogramming during NASH. Hepatocyte identity genes are downregulated, whereas genes involved in fibrosis and inflammation are induced during NASH.

The liver plays a critical role in the regulation of many metabolic functions, including lipid, carbohydrate and protein metabolism, glycogen storage, detoxification, bile production, and synthesis of plasma proteins. Metabolic-associated fatty liver disease (MAFLD) is a major health concern present in roughly 25% of the global population. MAFLD can further progress into non-alcoholic steatohepatitis (NASH) that is accompanied with increased hepatic fat accumulation (steatosis), inflammation, and fibrosis. A recent study [41] in mice fed a high fat diet (HFD) identified GLIS2 as a critical regulator of gene expression during the progression of MAFLD. ATAC-Seq analysis identified the *Glis2* gene as one of the genomic loci that obtained an open configuration during advanced NASH, whereas loci of genes associated with hepatic identity became closed. This correlated with a higher expression of GLIS2 in NASH. This study further showed that GLIS2 was one of the transcription factors that gained motif activity during NASH progression. These observations suggested a regulatory role for GLIS2 in the regulation of gene transcription during NASH progression (Figure 4A). This was supported by data showing that specific knock down of *Glis2* expression by adenovirus-associated virus (AAV) miRNA in hepatocytes from mice fed a HFD significantly reduced the expression of genes associated with extracellular matrix, inflammation, adhesion, and cell cycle, including *Col1a1*, *Tnf*, *Ctgf*, *Ccl2*, and *Adgre1* (Figure 4A). This was accompanied by a decrease in hepatic fibrosis, inflammation, and apoptosis; however, hepatic steatosis remained unaffected. GLIS2 binding sites (GLIS2BS) were associated with many of these differentially expressed genes indicating that their transcription is directly regulated by GLIS2. These data suggest that in this case, GLIS2 functions as an activator of gene transcription. The GLIS2BS in many of the target genes overlap with those of several other transcription factors that are implicated in NASH-associated gene expression, such as ELF3 and members of the AP-1 family. This suggests that GLIS2 regulates gene transcription of these genes in coordination with other transcription factors. Thus, GLIS2 together with other hepatic transcription factors are part of gene regulatory network that regulates NASH-associated gene expression in hepatocytes. The study further indicates that GLIS2 plays a critical role in promoting NASH-associated reprogramming in hepatic fibrosis, inflammation, and apoptosis; however, hepatic steatosis remained unaffected. GLIS2 binding sites (GLIS2BS) were associated with many of these differentially expressed genes indicating that their transcription is directly regulated by GLIS2. These data suggest that in this case, GLIS2 functions as an activator of gene transcription. The GLIS2BS in many of the target genes overlap with those of several other transcription factors that are implicated in NASH-associated gene expression, such as ELF3 and members of the AP-1 family. This suggests that GLIS2 regulates gene transcription of these genes in coordination with other transcription factors. Thus, GLIS2 together with other hepatic transcription factors are part of gene regulatory network that regulates NASH-associated gene expression in hepatocytes. The study further indicates that GLIS2 plays a critical role in promoting NASH-associated reprogramming in hepatocytes by repressing the expression of hepatocyte identity genes (e.g., *Cyp8b1*, *Aldh2*, *Idh1*, and *Slc2a2*) and increasing that of inflammatory and fibrosis-related genes (Figure 4B) [41]. These observations contrast those in kidney, in which loss of GLIS2 function induces inflammation and fibrosis [8,12,26,27]. These differential effects of GLIS2 in kidney and liver are likely due to tissue- and context-dependent differences.

## 7. Additional Roles for GLIS3 in Stem and Progenitor Cells

GLIS3 has several additional roles in the regulation of progenitor cell differentiation, renewal, and survival in several tissues, including the pancreas and testis [7,19,20]. In the pancreas, GLIS3 is most highly expressed in pancreatic β and ductal cells [6,7,22,23,24,99,100]. Loss of GLIS3 function causes neonatal diabetes that is, in part, due to a reduced generation of neurogenin 3-positive (NGN3^+^) endocrine progenitor cells during pancreatic development. GLIS3 directly regulates the expression of NGN3, a transcription factor required for the differentiation of bipotent progenitor cells into proendocrine and productal cells [101,102]. Reduced expression of NGN3 might be partly responsible for the decreased generation of NGN3^+^ pro-endocrine cells and endocrine cells in GLIS3-deficient pancreas. Whether this also involves effects on self-renewal or cell survival has yet to be established. GLIS3 also has important functions in postnatal pancreas, where it is essential for the transcriptional regulation of insulin genes (*Ins1* and *Ins2* in mice and *INS* in humans) expression and several other genes [7,22,23,24]. GLIS3 was shown to regulate *Ins2* transcription in coordination with other transcription factors [100]. Loss of GLIS3 function in both humans and mice also causes dilation of pancreatic ducts suggesting that GLIS3 might have a regulatory role in ductal cell homeostasis. A recent study demonstrated that *Glis3* is most highly expressed in CD133^+^CD71^−^ cells, a subpopulation of pancreatic ductal cells with high self-renewal capacity [103,104]. These progenitor cells are multipotent and able to differentiate along the acinar, ductal, and endocrine lineages [103]. *Glis3* knockdown reduces self-renewal in these cells that appears, at least in part, due to reduced transcription of *Prom1* (CD133). The stimulation of self-renewal in these cells by PROM1 appears to be mediated via the activation of PI3K/AKT and β-catenin pathways and induction of WNT signaling-related genes. These studies suggest that GLIS3 is required for the self-renewal of these progenitor cells by regulating *Prom1* expression [104]. This suggests that in addition to GLIS2, GLIS3 also has a connection with PROM1 and stem cell renewal.

GLIS3 is also essential for early spermatogenesis [19,43]. It is most highly expressed in pro-spermatogonia (ProSG; also referred to as gonocytes), spermatogonial stem and progenitor cells (SSCs and SPCs, respectively), but not at later stages of spermatogenesis. Shortly after birth, ProSGs resume proliferation and give rise to SSCs, which sequentially differentiate into SPCs and differentiated spermatogonia [105,106]. In addition to its function in regulating retrotransposon silencing [107], GLIS3 is involved in the control of cell proliferation and differentiation of SSCs and SPCs [19]. ProSGs, SSCs, and SPCs are significantly reduced in *Glis3*-deficient mice. This, subsequently, greatly impacts the generation of spermatozoa leading to infertility. The precise molecular mechanism underlying this regulation has yet to be determined.

GLIS3 has further been shown to regulate lineage determination of human pluripotent stem cells (hPSCs) into neural progenitor cells (NPCs) [40]. Addition of activin/TGFβ/BMP pathway inhibitors directs differentiation of hPSCs along the anterior NPC lineage that is associated by decreased expression of stem cell marker genes, including *OCT4*, *NANOG*, and *SOX2*, and induction of anterior NPC markers, such as *OTX1/2*, *ZIC1*, and *PAX6*. Expression of GLIS3 in hPSCs directs differentiation along the posterior NPC lineage in lieu of the anterior lineage as indicated by the induction of posterior NPC marker genes, such as *GBX2*, *MNX1*, and *HOXA2*. This study demonstrated that this differentiation along the posterior lineage was mediated by increased expression of *WNT* genes, particularly *WNT3A*, which encodes a strong posteriorizing factor [40,108,109]. GLIS3 was shown to activate *WNT3A* transcription directly by binding to a GLISBS in the *WNT3A* proximal promoter region.

## 8. GLIS Circular RNAs

In addition to linear RNAs required for the formation of GLIS proteins, *GLIS1-3* genes also generate CircGLIS RNAs. CircRNAs are lncRNAs that are generated through backsplicing of 1 or more exons that is facilitated by RNA binding proteins (RBPs). CircRNAs can localize to the nucleus and cytoplasm and can be secreted through exosomes [110,111]. They can regulate gene expression both at the transcriptional and post-transcriptional level. The most common mechanism of action is their interaction with miRNAs, thereby acting as miRNA sponges. CircRNAs can also modulate gene expression through their interaction with proteins, including RBPs. They can regulate various cellular functions, including EMT, stemness, apoptosis, senescence, and differentiation, and have been implicated in many cancers.

A recent study showed that the expression circular GLIS2 (circGLIS2) was significantly higher in colorectal cancer, the third most common cancer globally [112]. CircGLIS2 is generated from exon 2 and 3 via back-splicing. Over-expressing of circGLIS2 in human colorectal cell lines results in activation of the NF-κB signaling pathway by sponging miR-671, which results in the induction of several pro-inflammatory cytokines that, subsequently, increase cell migration and metastasis. The authors suggested that targeting circGLIS2 might provide a strategy to intervene in colorectal cancer [112].

A recent study reported that, in addition to circGLIS2, linear GLIS2 RNA also plays a role in colorectal cancer [29]. GLIS2 was identified as a moderate repressor of several p53 target genes, including BCL2 binding component 3 (*BBC3*, also referred to as *PUMA*), which encodes a pro-apoptotic protein. GLIS2 over-expression in human colon cancer HCT116 cells also repressed the expression of several focal adhesion genes. ChIP analysis indicated that GLIS2 was associated with the *BBC3* proximal promoter, a region where also p53 binds. Suppression of GLIS2 expression modestly increased p53 binding to this region. Subcutaneous injection of GLIS2-over-expressing HCT116 cells enhanced tumor load in mice and reduced their overall survival.

Recently, expression of circular GLIS3 (circGLIS3) RNAs, generated by exon 2 or exons 5–8, have been implicated in several tumor types, including glioma, non-small cell lung cancer (NSCLC), and bladder cancer [113,114,115]. Expression of circGLIS3, generated from exon 2, was found to be associated high-grade gliomas that are highly invasive and resistant to therapeutic treatments. Most of these gliomas consisted of glioblastoma, the deadliest type of brain cancer. Expression of this circGLIS3 enhances migration of glioma cells. Moreover, tumors derived from intracranial injection of circGLIS3-overexpressing glioma cells in nude mice were more invasive and reduced the overall survival of the mice [113]. CircGLIS3 was found to interact with T567-phosphorylated ezrin (p-EZR), but not with unphosphorylated ezrin, causing an increase in p-EZR, a protein that promotes cell migration and correlates with high-grade gliomas. This study further showed that circGLIS3 is secreted through exosomes and enhances angiogenesis. Thus, circGLIS3 appears to promote the malignancy of gliomas by both increasing invasiveness and angiogenesis. A different report showed that high levels of GLIS3 expression correlated with high-grade gliomas and poor prognosis [116]. Knockdown of GLIS3 expression in glioma cells lines reduced cell proliferation and migration, inhibited the activation of the NF-κB signaling pathway, and suppressed the in vivo malignant behavior of cells when implanted into nude mice. This correlated with respective changes in c-MYC, MMP9, and phosphorylated p65 expression. Over-expression of GLIS3 in glioma cell lines had the inverse effect.

CircGLIS3 containing exon 2 was also identified in bladder cancer tumors and shown to increase migration in cultured bladder cancer cells by absorbing miR-1273f resulting in increased expression of S-phase kinase protein 1 (SKP1) and cyclin D1 [114]. CircGLIS3 derived from exons 5–8 was found to be associated with cancer progression in NSCLC [115]. This circGLIS3 was shown to stimulate cell proliferation and migration and inhibit apoptosis in cultured NSCLC cells that appears to be mediated in part by the down-regulation of the tumor suppressor miR-644a. CircGlis3 RNAs appear to be also involved in the regulation of normal physiological processes, including pancreatic β cell function, as was recently reported for a circGLIS3 derived from exon 4 [117].

Together, these studies demonstrate that circGLIS2 and circGLIS3 enhance self-renewal and invasiveness in various tumor cell types. Whether this involves regulation of reprogramming, stemness, and EMT needs further study. Moreover, circGLIS RNAs may have a role in the regulation normal physiological functions as well. Thus, mutations or shRNA knockdown may affect the expression of linear *GLIS* RNAs and/or circRNAs and yield distinct phenotypes.

## 9. Conclusions

Evidence is accumulating that the transcriptional activity of GLIS proteins is regulated, at least in part, by primary cilium-dependent mechanisms. In addition, several studies have linked GLIS proteins to the regulation of cell reprogramming, cell lineage determination, and self-renewal. These processes play a critical role in normal mammalian development, while defects in their regulation are causally linked to many pathologies, including cancer. A better understanding of the molecular mechanisms by which GLIS proteins regulate gene transcription are critical for elucidating their function in the control of various biological processes and their roles in disease. Moreover, future insights into the signaling pathways that regulate the subcellular localization, post-translational modification, proteolytic processing, and transcriptional activity of GLIS proteins might lead to the development of new therapeutic strategies in the management of several pathologies.

## Data Availability

Not applicable.
